# The Role of Cannabinoid Transmission in Emotional Memory Formation: Implications for Addiction and Schizophrenia

**DOI:** 10.3389/fpsyt.2014.00073

**Published:** 2014-06-30

**Authors:** Huibing Tan, Tasha Ahmad, Michael Loureiro, Jordan Zunder, Steven R. Laviolette

**Affiliations:** ^1^Department of Anatomy and Cell Biology, Schulich School of Medicine and Dentistry, University of Western Ontario, London, ON, Canada; ^2^Department of Psychiatry, Schulich School of Medicine and Dentistry, University of Western Ontario, London, ON, Canada; ^3^Department of Psychology, Schulich School of Medicine and Dentistry, University of Western Ontario, London, ON, Canada

**Keywords:** cannabinoids, emotion, schizophrenia, addiction, opiates, frontal cortex, dopamine, amygdala

## Abstract

Emerging evidence from both basic and clinical research demonstrates an important role for endocannabinoid (ECB) signaling in the processing of emotionally salient information, learning, and memory. Cannabinoid transmission within neural circuits involved in emotional processing has been shown to modulate the acquisition, recall, and extinction of emotionally salient memories and importantly, can strongly modulate the emotional salience of incoming sensory information. Two neural regions in particular, the medial prefrontal cortex (PFC) and the basolateral nucleus of the amygdala (BLA), play important roles in emotional regulation and contain high levels of cannabinoid receptors. Furthermore, both regions show profound abnormalities in neuropsychiatric disorders such as addiction and schizophrenia. Considerable evidence has demonstrated that cannabinoid transmission functionally interacts with dopamine (DA), a neurotransmitter system that is of exceptional importance for both addictive behaviors and the neuropsychopathology of disorders like schizophrenia. Research in our laboratory has focused on how cannabinoid transmission both within and extrinsic to the mesolimbic DA system, including the BLA → mPFC circuitry, can modulate both rewarding and aversive emotional information. In this review, we will summarize clinical and basic neuroscience research demonstrating the importance of cannabinoid signaling within this neural circuitry. In particular, evidence will be reviewed emphasizing the importance of cannabinoid signaling within the BLA → mPFC circuitry in the context of emotional salience processing, memory formation and memory-related plasticity. We propose that aberrant states of hyper or hypoactive ECB signaling within the amygdala-prefrontal cortical circuit may lead to dysregulation of mesocorticolimbic DA transmission controlling the processing of emotionally salient information. These disturbances may in turn lead to emotional processing, learning, and memory abnormalities related to various neuropsychiatric disorders, including addiction and schizophrenia-related psychoses.

## Functional Roles for Cannabinoid Signaling in Neural Emotional Processing Centers

The endocannabinoid (ECB) system is widely distributed in the mammalian brain and demonstrates relatively high density in neural regions implicated in the processing of emotionally salient information, such as the basolateral nucleus of the amygdala (BLA), prefrontal cortex (PFC), VTA, and NAc (1, 2). Naturally occurring ECBs include 2-arachidonoylglycerol (2-AG) and anandamide, which produce their actions via distinct neurophysiological mechanisms. Thus, while 2-AG is known to act as a fast-acting and transient retrograde messenger, anandamide can produce slower retrograde synaptic effects (3). In both cases, the canonical understanding of ECB function is that ECBs are generally released from post-synaptic neuronal elements and feedback in a retrograde manner onto pre-synaptic terminals, which may be either excitatory or inhibitory in nature (4). Retrograde synaptic modulation via the ECB system is known to be involved in short-term synaptic depression and can suppress both excitatory and inhibitory signaling within specific neuronal circuits (3, 4). As will be described presently, given this anatomical and pharmacological complexity, a wide variety of potential functional consequences can be attributed to CB1 receptor activation (via either ECBs, plant-derived or synthetic cannabinoid receptor agents). For within the BLA, CB1 receptors are localized primarily on inhibitory local GABAergic interneurons or terminals, but are absent in the adjacent central nucleus (1, 5, 6). In this case, activation of BLA CB1 receptors is known to decrease feedforward inhibition via inhibitory interneurons thereby increasing the activity of BLA projection neurons (7), leading to modulatory effects in efferent projection targets, such as the PFC (8, 9). Conversely, activation of CB1 receptors associated with excitatory pre-synaptic elements, such as glutamatergic terminals, may be capable of producing net inhibitory post-synaptic effects, depending upon the specific neuronal circuit under investigation. In both cases, emerging evidence points to the primary functional role of ECB signaling as indirectly modulating neuronal outputs via actions on pre-synaptic elements, primarily involving the inhibition of feedforward inhibitory influences on these neuronal outputs. As will be reviewed below, such mechanisms implicate the ECB system as a critical functional relay capable of controlling the output of a variety of neural emotional processing centers, including connections between the BLA and PFC, and the regulation of DAergic signaling within the mesocorticolimbic circuit.

## Evidence for Dysregulated Endocannabinoid Signaling in Schizophrenia and Co-Morbid Addictive Behaviors

Abnormal emotional processing, learning and memory are core features of various neuropsychiatric conditions, including schizophrenia and addiction. Disturbances in the ability to form appropriate associative memories and perform adaptive associative learning tasks, has been identified as critical neuropsychopathological features in both schizophrenia (10, 11) and addiction (12, 13). In both cases, patients may be more likely to inappropriately assign distorted emotional significance to internal or external stimuli, leading to aberrant associative memory formation. Drug-dependent individuals, including those presenting substance abuse co-morbidly with other neuropsychiatric disorders, attribute pathologically elevated motivational salience to drug-predictive cues, leading to compulsive drug seeking and relapse. Individuals with schizophrenia may attribute inappropriate emotional salience (either appetitive or aversive) to events or stimuli within the environment that healthy individuals would dismiss as non-significant, ultimately leading to delusional beliefs and/or psychotic ideation. In addition, substance abuse is highly co-morbid with schizophrenia-related psychopathology (14). Signaling through the brain’s ECB system is involved in a plethora of behavioral, synaptic, and neuronal phenomena associated with these processes, and is a critical modulator of both rewarding and aversive emotionally salient information. However, emerging evidence from both human and basic neuroscience research, points to the importance of functional interactions between ECB signaling, and sub-cortical transmission through the dopamine (DA) system, as a nexus point to understanding how dysregulation of the ECB system may relate to the well-established aberrations in DAergic transmission, as a core neuropathological feature of both addiction and schizophrenia. Indeed, mounting evidence implicates disturbances in neural reward pathways as underlying neuropathological features of schizophrenia, indicated by abnormal blunting of neural response patterns following exposure to normally rewarding stimuli (15–17). This evidence suggests a core underlying disturbance in schizophrenia that transcends emotional valence, comprising deficits in the processing of both rewarding, appetitive information, along with aversive, negative emotional stimuli.

Considerable evidence from both human clinical and animal modeling studies points to ECB-dependent mechanisms that may be related to increased vulnerability to addictive behaviors and schizophrenia-related psychopathology. Interestingly, these disturbances have been associated both with genetic factors and have also been demonstrated following acute or chronic exposure to drugs that directly interact with the brains ECB systems, most notably, cannabis. In terms of characterizing the dysregulation of ECB signaling in schizophrenia-related symptomology, a land-mark paper from Andréasson et al. (18) provided compelling evidence suggesting that exposure to marijuana during critical, adolescent windows of neurodevelopment was positively correlated with an increased propensity to develop schizophrenia-related psychoses in early adulthood. Examining a cohort of 45,570 Swedish military conscripts, this report described a significant association between exposure levels to cannabis use in adolescence and the later emergence of schizophrenia reported during a 15-year follow-up. The authors reported that the relative risk for developing schizophrenia among heavy users of marijuana (>50 exposures) was 6.0 (95% confidence interval), compared with non-users. Importantly, this association was reliable after controlling for the presence of other neuropsychiatric diseases and social background, demonstrating that relative adolescent marijuana use represented an independent risk factor for the later development of schizophrenia. Andréasson et al. followed up this initial report with a subsequent longitudinal study using a sub-group from the original cohort (19). Comparing clinical records for schizophrenia-diagnosed subjects reporting marijuana use on more than 10 occasions vs. subjects who had gone on to develop schizophrenia in the absence of any reported marijuana exposure, still revealed a relative risk factor for schizophrenia among the marijuana-using sub-group as 4.1 (95% confidence interval). Causal roles for other narcotic drug exposures in the marijuana-using sub-group were ruled out. Intriguingly, the authors described a unique pattern of psychopathology among the marijuana users, characterized with a more acute onset of schizophrenia-related symptoms relative to non-exposed control subjects.

In addition to longitudinal relationships between marijuana exposure and the etiology of schizophrenia, considerable evidence points to dysregulation of naturally occurring ECBs as correlative factors linked to the occurrence of schizophrenia. Thus, several reports have shown that cerebrospinal fluid (CSF) levels of anandamide, a naturally occurring ECB, in medication-naive patients with schizophrenia are significantly elevated and negatively correlated with the presence of psychotic symptoms (20–23). Analysis of post-mortem brain tissue samples from schizophrenia patients revealed that the levels 2-AG, were significantly elevated relative to controls in several neural regions including the cerebellum, hippocampus, and PFC (24). Further evidence implicating a specific role for prefrontal cortical cannabinoid dysregulation as an etiological mechanism in schizophrenia has come from post-mortem studies of schizophrenia brain samples. An important study from Dalton et al. (25) analyzed cannabinoid CB(1) receptor binding levels and mRNA expression in the dorsolateral prefrontal cortex (DLPFC), comparing sub-populations of paranoid or non-paranoid schizophrenia patients, relative to healthy controls. Interestingly, this study reported a paranoid schizophrenia-subtype specific effect with paranoid schizophrenia subjects displaying 22% higher levels of CB1 receptor binding compared to controls or non-paranoid schizophrenia patients. Similar results were reported by Jenko et al. (26), wherein CB1 receptor binding in the DLPFC was reported to be 20% higher in schizophrenia relative to healthy control subjects. Similarly, Zavitsanou et al. (27) reported increased CB1 binding levels in samples of anterior cingulate cortex from schizophrenia patients. Such findings strongly suggested that abnormalities in CB1 receptor signaling within the PFC may relate specifically to paranoia-related schizophrenia symptomology and more specifically, suggest that a hyperactive state of CB1 activity within frontal cortical regions may be linked to the emotional regulation disturbances found in paranoid schizophrenia populations.

Beyond its well-established role in the processing of aversive emotional information and memory encoding, considerable evidence links the ECB system to the regulation of reward-related processing, specifically in the context of the opiate-receptor system. From a genetic perspective, genes encoding both the CB1 receptor (CNR1) and fatty acid amide hydrolase (FAAH) responsible for the breakdown of anandamide are located on chromosomes 6 and 1 in the 6q15 and 1p33 cytogenetic bands. Several reports have suggested that polymorphic mutations in these genes may be linked to addictive vulnerability to several drugs of abuse, including alcohol (28), nicotine (29), cocaine (30), and heroin (31). Cannabis use and exposure has long been theorized to represent a “gateway” drug, associated with an increased propensity to use other drugs of abuse, either concomitantly, or following long-term cannabis use. Such theories remain highly controversial for a number of reasons. First, it is virtually impossible to control for strain variety of cannabis exposure, given that cannabis strains may differ markedly in terms of relative THC or other chemical constituents. Second, obtaining documented and reliable histories of cannabis exposure that rely on self-report are difficult to acquire. Nevertheless, while such a “gateway” role for cannabis exposure has not been causally demonstrated in human, clinical populations, considerable evidence from basic neuroscience research, demonstrates a causal role for CB1 signaling in modulating sensitivity to the rewarding and/or dependence-producing properties of other drug classes, particularly opiate-class drugs, such as morphine and heroin, as will be discussed presently.

Despite an abundance of clinical evidence linking disturbances in prefrontal cortical cannabinoid signaling to schizophrenia and addiction-related psychopathology, the precise mechanisms related to these effects are not currently understood. However, research from basic neuroscience studies using behavioral and neuronal models of emotional processing and memory formation, point to several important functional circuits and mechanisms, which may underlie cortical CB1-mediated modulation of emotional processing. Specifically, considerable evidence now suggests important functional interactions between intra-cortical cannabinoid receptor substrates with sub-cortical DA transmission, and via cross-talk with the BLA.

## Cannabinoid Modulation of Emotional Processing, Learning, and Memory in the Amygdala-Prefrontal Cortical Pathway

Considerable evidence implicates the importance of functional communication within the BLA → PFC pathway as a mediator of emotionally salient learning, memory, and synaptic plasticity. For example, acute exposure to stressful/aversive events triggers long-term potentiation (LTP) or long-term depression within the BLA → PFC circuit, respectively (32, 33). Single neurons within the rodent mPFC show associative firing and bursting response patterns during fear-related learning, through a BLA-dependent input pathway (34). Similarly, neurons within the rodent BLA can demonstrate associative neuronal responding to odor cues paired with aversive stimuli such as foot-shock (35). In both cases, associative neuronal responding to foot-shock-paired olfactory cues can be demonstrated under full anesthesia, and are dependent upon DA transmission (34–37). In terms of cannabinoid modulation of emotional learning and memory processing within the BLA → PFC pathway, systemic administration of synthetic agonists of CB1 receptors, such as WIN 55, 212-2, can potently increase associative neuronal responding to fear-paired associative cues, both by increasing neuronal firing frequency and by increasing bursting activity of PFC neurons following presentations of associative CS+ cues (38). In contrast, blockade of CB1 transmission with CB1 receptor antagonists, were shown to completely block the ability of PFC neurons to encode associative neuronal responses to stimuli paired with foot-shock, demonstrating for the first time, bi-directional modulation of fear memory acquisition by activation or blockade of CB1 receptor transmission directly in the PFC. These bi-directional neuronal associative effects could be similarly demonstrated behaviorally; using an olfactory fear conditioning assay in awake, behaving rats, direct intra-PFC CB1 receptor activation strongly potentiated fear memory formation by increasing the emotional salience of normally non-salient fear memories, using a sub-threshold foot-shock conditioning stimulus. In contrast, blockade of intra-PFC CB1 transmission blocked the formation of supra-threshold fear memory formation. Importantly, these effects were not related to any changes in sensory or physiological, nociceptive sensitivity to the experience of the foot-shock stimulation (38).

In the context of emotional memory formation, the PFC and BLA represent a highly interconnected circuit, sharing functional ascending and descending connections. Neurons within the BLA are modulated by PFC inputs, with inputs from the PFC capable of suppressing BLA output neuron activity and sensory evoked excitation of BLA neurons, through DA-dependent mechanisms (35, 37). In addition, as described previously, emotional experience strongly modulates synaptic plasticity along the BLA → PFC circuit (32, 39). In terms of CB1 receptor modulation of the BLA and PFC, both regions show wide distribution of CB1 receptors, which have been demonstrated to modulate both the release of glutamate from pre-synaptic terminals and γ-aminobutyric acid (GABA) from local interneuron inhibitory elements (40–43). Thus, cannabinoids acting within either region are capable of modulating the activity of output neuronal populations and exerting effects distally in target regions, including the mesolimbic DA pathway. For example, cannabinoid compounds strongly modulate excitatory outputs from the PFC and BLA to neurons within the NAc (7, 44). Systemic cannabinoid drug administration also increases excitability of PFC neurons to VTA DAergic inputs (45) and can indirectly increase VTA DA neuron excitability by inhibiting GABAergic inhibition directly within the VTA (46).

## Cannabinoid Signaling in the BLA > PFC Pathway: Modulatory Effects on Emotional Processing and Memory

The amygdala is perhaps the most fully characterized neural region involved in the processing of emotionally salient information, including both reward-related and aversive fear memories (34, 36, 47–49). High levels of CB1 receptors are localized in the BLA region of the amygdala (2, 6) and CB1 transmission within the BLA is involved in the regulation of associative learning and memory processing (50, 51). A wealth of evidence has demonstrated that dysregulated emotional processing in patients with schizophrenia is associated with abnormal activity patterns within the amygdala. For example, distortions in the perception of emotional valences associated with pictures of human faces showing a range of emotion-related expressions is correlated with hyperactive responses in the amygdala region of human schizophrenia patients (52–54). Schizophrenia patient’s show markedly reduced amygdala volumes (55) and these abnormalities are correlated with specific stages of psychosis (56). Cannabinoids produce both inhibitory and excitatory effects within BLA neuronal sub-populations through both CB1 and non-CB1 receptor substrates (7). Nevertheless, the precise mechanisms by which cannabinoids may control neuronal output activity from the BLA is not entirely clear.

**Figure 1:**
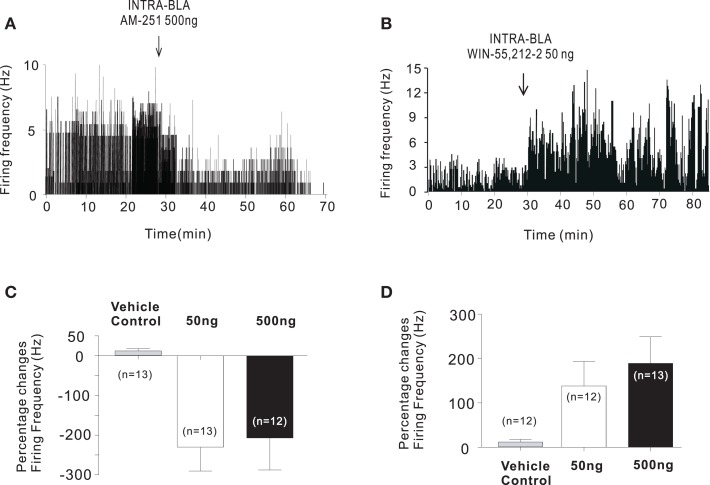
**Intra-BLA CB1 receptor modulation controls the activity of PLC neurons**. **(A)** Microinfusions of the CB1 antagonist AM 251 (50–500 ng/0.5 μl) strongly decreased the spontaneous firing frequency of a plurality of recorded PLC neurons. **(B)** In contrast, microinfusions of the CB1 agonist WIN 55, 212-2 (50–500 ng/0.5 μl) strongly increased the spontaneous firing frequency of a plurality of recorded PLC neurons. **(C)** Summary of group PLC neuronal data showing inhibitory effects on PFC neuronal activity following intra-BLA infusions of AM 251. **(D)** Summary of group PFC neuronal data showing excitatory effects on PLC neuronal activity following intra-BLA infusions of WIN 55, 212-2. Adapted from Tan et al. (9).

Given the well-established relationships between the BLA and PFC regions, we examined the effects of CB1 receptor modulation directly within the BLA on the acquisition of fear memory and on the modulation of distal neuronal activity in the prelimbic (PLC) division of the rat PFC. Following bilateral activation or blockade of intra-BLA CB1 receptors, using WIN 55, 212-2, or AM-251, respectively, we found that CB1 receptor activation within the BLA could strongly amplify the salience and promote the formation of associative olfactory fear memories to normally non-salient, sub-conditioning threshold levels of foot-shock (9). In contrast, blockade of CB1 transmission with a CB1 receptor antagonist completely blocked the formation of associative fear memories to normally supra-threshold levels of foot-shock. Concomitantly, these same behaviorally effective doses of intra-BLA CB1 receptor agonists or antagonists were able to bi-directionally control the spontaneous neuronal activity patterns of sub-populations of isolated neuronal units recorded *in vivo*, in the PLC; whereas intra-BLA CB1 receptor activation caused a strong increase in a plurality of recorded PLC neurons, blockade of CB1 receptors caused a predominant decrease in these neuronal activity levels (Figure 1). In addition to the effects induced by direct pharmacological CB1 receptor blockade or activation, we found that intra-BLA administration of AM 404, a synthetic anandamide transport inhibitor and transient receptor potential cation channel subfamily V member 1 (TrpV1) receptor agonist, mimicked the effects of direct CB1 receptor activation with WIN 55, 212-2, suggesting that intra-BLA cannabinoid modulation of emotional memory processing is dependent upon regulation of a tonic CB1 receptor-mediated pattern of activation. Together, such findings are consistent with prior evidence demonstrating that (1) neurons in the PFC involved in the encoding of associative fear memories are comprised of a sub-population of cells that receive inputs from the BLA (34) and (2) the ability of CB1 transmission within the PFC to modulate emotional memory formation depends upon functional inputs from the BLA (38). Further evidence also implicates the importance of this pathway in cannabinoid-mediated modulation of emotional memory formation. For example, Campolongo et al. (57) reported that infusions of the CB1 agonist WIN 55, 212-2 into the rodent BLA was able to enhance memory retention during an inhibitory avoidance task, while blockade of CB1 transmission produced the opposite effect. Interestingly, these effects were dependent upon glucocorticoid transmission, suggesting that intra-BLA modulation of emotional memory processing interacts with stress-related signaling substrates. Such findings are consistent with evidence demonstrating that attenuation of ECB signaling within the BLA is involved in the activation of the stress-response via the hypothalamic–pituitary–adrenal axis (58), which further implicates the importance of intra-BLA CB1 transmission as a critical regulatory component for the processing of emotionally salient experiences.

## Cannabinoid Signaling in the BLA → PFC Pathway: Integrative Control of Emotional Processing and Memory-Related Plasticity

Considerable evidence links modulation of cannabinoid signaling within the mammalian PFC as a critical modulator of emotional processing and memory formation. Exposure to stressors has been reported to increase expression levels of CB1 receptors directly within the PFC (59) and, as noted previously, considerable evidence, particularly from post-mortem analyses of brain tissue from schizophrenia patients, has revealed pathological dysregulation of CB1 receptor signaling in PFC regions. We have reported that direct activation of CB1 receptor transmission in the rodent PFC can potentiate the formation of associative fear memories to cues paired with normally sub-threshold, non-salient levels of foot-shock. Thus, associative responses to olfactory cues paired with aversive foot-shock stimulation are strongly potentiated in neuronal sub-populations recorded in the rat PFC (38). This CB1-mediated potentiation in associative neuronal fear memory encoding is reflected both in terms of increased associative bursting and firing frequency rates. Importantly, the ability of intra-PFC CB1 receptor activation to potentiate emotional salience and memory formation depends upon functional inputs from the BLA as PFC neurons demonstrating potentiated emotional memory formation represent a sub-population of PFC neurons that respond to discrete, orthodromic electrical stimulation of the BLA. Furthermore, reversible pharmacological inactivation of the BLA blocked the emotional salience potentiating effects of intra-PFC CB1 receptor activation (38).

**Figure 2:**
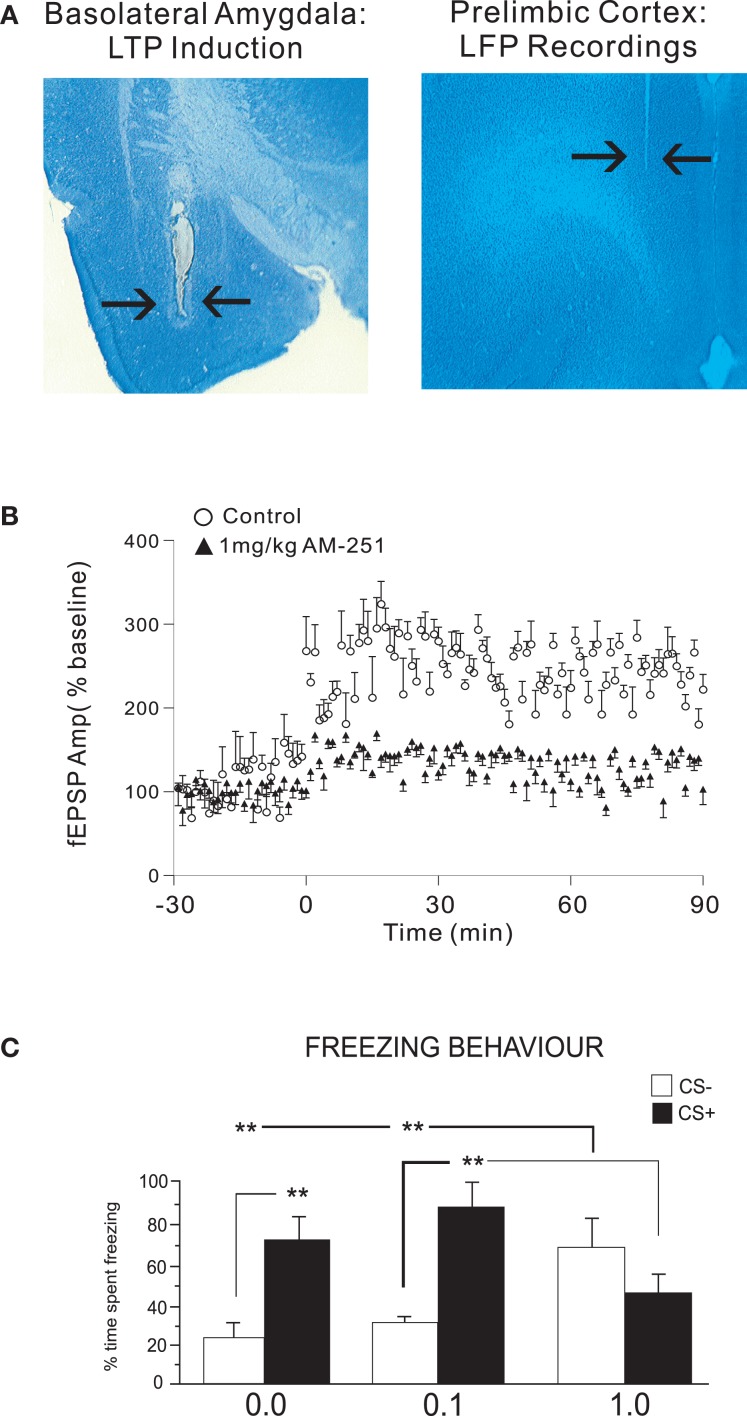
**Cannabinoid transmission controls synaptic plasticity and fear memory formation in the BLA → PFC pathway**. Effects of systemic AM-251 pre-treatment on the induction of *in vivo* LTP along the BLA → PLC pathway. **(A)** Using an *in vivo* LTP induction protocol in rats, we recorded local field potentials in the PLC following the induction of LTP following tetanic, electrical stimulation of the BLA. **(B)** Group data comparing excitatory post-synaptic potential (EPSP) amplitudes from animals receiving systemic injections of the CB1 receptor antagonist, AM 251 (1.0 mg/kg; i.p.) vs. saline vehicle controls. AM 251 pre-treatment completely prevented the induction of LTP along the BLA → PLC pathway. **(C)** Remarkably, this same dose of systemic AM 251 (1.0 mg/kg; i.p.) completely blocked the acquisition of fear memory in awake, behaving rats, as measured by freezing behaviors following presentations of fear-associated olfactory cues; **p < 0.01; *p < 0.05.

Given the bi-directional effects of CB1 receptor modulation within the BLA → PFC pathway in the neuronal and behavioral processing of emotional memory, we further examined how associative plasticity in the form of LTP within the BLA → PFC pathway may be modulated by CB1 transmission. While previous reports have demonstrated that intra-PFC CB1 signaling can modulate glutamatergic synaptic plasticity mechanisms (60), it was not known how CB1 signaling may be involved in the mediation of synaptic plasticity along the BLA → PFC pathway. After adapting an *in vivo* electrophysiological protocol for inducing LTP within the PFC following tetanic stimulation of the BLA first reported by Maroun and Richter-Levin (39), we examined the effects of pharmacological blockade of CB1 receptors on the induction of BLA → PFC LTP (Figure 2A), via systemic administration of AM 251, prior to the induction of LTP in anesthetized rats (8). Consistent with evidence implicating a functional relationship between CB1 transmission in the BLA → PFC pathway during associative learning and memory processing, we found that CB1 receptor blockade completely blocked the induction of LTP within the BLA → PFC circuit (Figure 2B). Interestingly, this same systemic dose of AM 251 was sufficient to completely block the formation of associative fear memories in awake, behaving rats (Figure 2C). Furthermore, functional disconnection experiments performed by contralateral blockade of CB1 transmission in the BLA or PFC, revealed that the acquisition of fear memory within this pathway required simultaneous CB1 receptor activation in both regions. Thus, the acquisition of associative fear memory within this circuit requires integrative CB1 receptor signaling, consistent with the known functional interconnections between the BLA and PFC. While this report was the first to demonstrate a functional role for CB1 signaling in the development of LTP within the BLA → PFC pathway, these findings are consistent with a large body of evidence implicating the ECB system in the modulation of learning and memory-related synaptic plasticity mechanisms in other neural regions, particularly the hippocampus, wherein signaling through CB1 receptor substrates is known to modulate associative synaptic plasticity processes (61, 62). Thus, consistent with the known disturbances in ECB signaling in PFC regions and the well-established deficits in emotional processing within the amygdala-PFC circuit in patients with schizophrenia (63), these findings demonstrate that appropriate emotional processing and memory formation within the BLA → PFC pathway requires integrative CB1 transmission across this circuit. Hyper- or hypo-activation of CB1 receptor substrates within either region are sufficient to cause pathological amplification of normally non-salient emotional stimuli, or, a blunting of emotional salience toward environmental stimuli that would normally produce adaptive associative memories and learned behaviors (8, 38).

## Cannabinoid CB1 Receptor-Mediated Modulation of Reward Processing: Interactions with Opiate Reward Signaling

The brain’s naturally occurring opiate-receptor system and associated neural circuitry represents an ideal experimental system to examine how ECB transmission may modulate motivational states and memory formation. Indeed, opiate-class stimuli, either pharmacological formulations or naturally occurring opiate peptides, serve as powerful reinforcers and conditioned stimuli during a variety of reward-related learning and memory tasks in humans and other animals. Considerable evidence using behavioral genetic approaches has demonstrated several important functional interrelationships between ECB and opiate-related reward processing, both in terms of opiate-related reward behaviors and in terms of CB1 receptor-mediated modulation of neural regions critical for opiate reward processing, such as the mesocorticolimbic system. Early behavioral reports using genetic knock-out (KO) mutant mice models of the CB1 receptor showed marked reductions in heroin intravenous self-administration (64, 65). Interestingly, the blunting of heroin self-administration in the CB1 KO mice appeared to be specific to opiates (64). The mammalian VTA is a critical neural substrate for the processing of the primary rewarding properties of opiates, through DA-dependent and non-DA neuronal substrates (66, 67) and a large body of evidence has demonstrated that cannabinoid compounds can acutely activate both the opiate and DAergic signaling systems. For example, the acute administration of THC has been reported to increase levels of β-endorphins and enkephalinergic peptides directly in the mesolimbic DA pathway, including the VTA and NAcc (68). Acute THC also increases the reinforcing efficacy of intravenously administered heroin (69) facilitates the release of DA from NAc DAergic terminals (70, 71) and directly activates VTA DA neurons recorded *in vivo* (72). In rat neurodevelopmental models, adolescent THC exposure has been reported to strongly increase opiate self-administration in early adulthood (73) and potentiate mesolimbic DA release (74). Functionally, this effect has been linked to THC-induced up-regulation of the pro-enkephalin peptide, directly in the shell region of the nucleus accumbens [NAc; (75)]. Such findings following adolescent THC exposure, similar to those reported with schizophrenia-related emotional and behavioral abnormalities, further implicate the importance of THC exposure during particular neurodevelopmental windows, which in turn influence later adulthood phenotypes. Nevertheless, beyond the actions of CB1 transmission on DAergic signaling within the mesolimbic system, it is currently not understood how CB1 transmission modulates reward processing via interactions with regions extrinsic to the mesolimbic system, such as the PFC and BLA, nor how such systems level interactions may control sensitivity to the rewarding and dependence-producing properties of opiate-class drugs of abuse.

## Cannabinoid Modulation of Mesocorticolimbic Activity: Implications for Reward Processing Dysregulation in Psychiatric Disorders

Beyond the VTA and NAc, prefrontal cortical regions are involved importantly in the modulation of opiate reward salience. Specifically, the PLC division of the mammalian PFC has been demonstrated to provide modulation of morphine-related reward processing and neuronal populations within the PLC are critically involved in the acquisition, expression, and extinction of opiate-related reward learning and memory (48, 76, 77). For example, using *in vivo* multi-unit recordings in behaving rats, we have demonstrated previously that sub-populations of neurons within the PLC actively encode associative memories related to the rewarding effects of morphine, as measured in the conditioned place preference (CPP) procedure, a Pavlovian conditioning model of drug seeking behaviors (76). Similar to the role of PFC neurons in the encoding of aversive, fear-related information, the neuronal encoding of reward-related information in the PLC is controlled by functional input from the BLA (49). Given these dual roles for PLC neuronal sub-populations in the processing of both aversive, fear-related information (34, 36, 38) and rewarding, appetitive opiate-related learning and memory (48, 76), we examined how direct modulation of CB1 receptor signaling within the PLC may modulate the processing of opiate-related reward information, and how CB1 transmission directly within the mammalian PLC may modulate opiate reward transmission via outputs to the VTA and mesolimbic system (78).

Using an unbiased CPP procedure in rats, we performed bilateral microinfusions of either a direct CB1 receptor agonist (WIN 55, 212-2) or antagonist (AM 251), prior to behavioral conditioning sessions using either a highly rewarding, supra-threshold conditioning dose of morphine (5 mg/kg; i.p.) or a sub-threshold, non-rewarding conditioning dose of systemic morphine (0.05 mg/kg), based upon previously established and reported behavioral dose-response curves for morphine (47, 48). Surprisingly, we found that blockade of CB1 transmission directly within the PLC division of the PFC, strongly potentiated the reward salience of normally sub-reward threshold conditioning effects of morphine while having no effects on the rewarding behavioral properties of normally supra-threshold, highly rewarding doses of morphine. These results indicated that rather than blunting the motivational salience of morphine, blockade of CB1 transmission directly in the PLC was able to potentiate the reward salience of sub-threshold reward signals. In stark contrast, direct activation of CB1 receptors within the PLC produced the opposite pattern of behavioral results; switching the motivational valence of a normally highly appetitive conditioning dose of systemic morphine from rewarding, to aversion, with rats demonstrating strong conditioned place aversions (CPA) to environments paired previously with supra-threshold doses of morphine (78). Given previous reports that direct activation of CB1 transmission in the PFC or BLA is able to strongly potentiate the aversive salience of normally non-salient conditioning levels of foot-shock (9, 38) while blocking CB1 transmission is capable of blocking the acquisition of salient associative fear memories, these results are seemingly incongruent with a role for hyperactive intra-PFC CB1 receptor substrates as an amplification mechanism for emotionally salient information. Furthermore, these opposite effects of intra-PFC CB1 receptor activation vs. blockade on processing of reward-related associative memory may suggest that the functional role of intra-PLC CB1 receptor transmission, is capable of producing bi-directional modulation of aversive or rewarding emotional information in opposite directions, depending on the motivational valence of the conditioning events (e.g., fear vs. reward based learning assays). However, an alternative explanation is that hyper-activation or CB1 receptor transmission within the PFC may amplify specifically negative aspects of associative learning stimuli. In this case, sub-threshold fear-inducing stimuli would be behaviorally potentiated in the fear learning paradigm, while the aversive stimulus properties of morphine would similarly be amplified and expressed as CPA, as measured in place conditioning procedures (78). Indeed, as will be discussed presently, morphine possesses powerful aversive stimulus effects, in addition to its potent rewarding properties (79, 80). Thus, intra-PFC CB1 receptor hyper-stimulation can amplify aversive emotional information whilst concomitantly switching reward signals into aversion. In contrast, hypoactive states of intra-PFC CB1 activity can blunt the salience of normally aversive emotional events, while concomitantly potentiating the reward salience of normally sub-reward threshold stimuli. Interestingly, both effects are mediated through DA-dependent transmission as systemic blockade of both DA D1 and D2 transmission with a broad-spectrum DA receptor antagonist was sufficient to block both the reward potentiating effects of intra-PLC receptor blockade, and the aversion-inducing properties of intra-PLC CB1 receptor activation, suggesting that the final common output for intra-PFC mediated modulation of opiate-related reward signaling is through a DAergic mechanism.

As with many drugs of abuse, opioid compounds, including morphine, possess both rewarding and aversive stimulus properties that can be demonstrated in a variety of behavioral assays (47, 67, 79–81). Following the demonstration that intra-PLC CB1 receptor modulation could switch the motivational valence of opiates from reward to aversion or potentiate the motivational salience of normally sub-reward threshold doses of morphine, we examined which specific opiate-receptor substrates within the VTA may mediate these effects. Several pieces of evidence demonstrate that the rewarding or aversive stimulus properties of opiates can be dissociated via separate μ-opiate-receptor (MOR) vs. κ-opiate-receptor (KOR) substrates within the VTA. For example, while transmission via MOR receptors is associated with indirect activation of DA neurons (82, 83), activation of KOR substrates in the VTA is linked to aversive opiate effects (80) and to the inhibition of VTA DAergic neuronal populations (84, 85). Given these dissociable neuronal and behavioral effects of MOR vs. KOR receptor populations, we hypothesized that the ability of intra-PLC CB1 receptor blockade or activation to modulate opiate-related motivational signals may depend upon signaling through these separate receptor substrates directly in the VTA. Consistent with this hypothesis, we found that the ability of intra-PLC CB1 receptor activation to switch a normally rewarding dose of morphine into an aversive behavioral effect was dependent on a KOR signaling mechanism directly in the VTA. In contrast, the ability of intra-PLC CB1 receptor blockade to potentiate the rewarding salience of normally sub-reward threshold conditioning doses of morphine was mediated through a MOR-dependent mechanism, directly in the VTA. Currently, the precise mechanisms by which intra-PFC CB1 activation or blockade may differentially activate either a MOR-dependent opiate reward system vs. a KOR-dependent aversion signaling pathway are not known. However, given that both behavioral phenomena are dependent upon DAergic signaling (78), it is likely that there is a convergence of these motivational effects via DA receptor substrates emerging from the VTA. One possibility comes from anatomical evidence from Ford (86), who reported that VTA KOR-associated DA neuron sub-populations preferentially projected to NAc targets whereas VTA MOR-associated sub-populations showed preferential targeting to the BLA. Interestingly, the NAc has been implicated in mediating DA-dependent aversive motivational effects, such as those associated with nicotine (87, 88). Furthermore, intra-BLA DA receptor transmission has been demonstrated to strongly modulate the rewarding properties of opiates (47, 89). In this case, the aversive or rewarding properties associated with opiate conditioning could be mediated through DA receptor-dependent substrates, but via anatomically distinct projections. Future studies are required to more precisely determine the functional relationships between these VTA output target pathways and how bi-directional CB1 signaling within the PFC may differentially trigger these systems via descending influences on VTA MOR vs. KOR signaling substrates. Thus, via direct interactions with sub-cortical DAergic motivational systems within the VTA, cortical CB1 receptors are capable of modulating reward processing, and even switching the emotional valences of normally highly rewarding conditioning stimuli, such as morphine, into aversive behavioral effects (78).

Beyond the modulation of opiate-receptor-mediated motivational effects, a recent study from Katsidoni et al. (90) reported that administration of THC was capable of bi-phasically modulating reward processing and behavioral activation as measured in a rewarding electrical brain stimulation paradigm involving self-stimulation of the medial forebrain bundle. While lower THC doses were shown to enhance generalized reward processing, higher doses caused blunted reward activity. Furthermore, beyond the opiate-receptor system, a large body of research implicates a role for CB1 transmission in the modulation of non-drug, natural reward stimuli such as food reinforcers. For example, Hernandez and Cheer (91) reported that blockade of CB1 transmission could blunt the rewarding properties of natural food reinforcers and concomitantly block neuronal reward-related signaling in neurons recorded from the NAc. Together, such evidence demonstrates a more generalized role for CB1 receptor-mediated modulation of reward information within the mesocorticolimbic system that can transcend drug-related motivational processing. Future studies will likely reveal more specific mechanisms related to different classes of rewarding stimulation and how CB1-mediated transmission may differentially influence separate classes of behavioral reinforcers.

While further studies are required to precisely identify how intra-PLC CB1 receptor signaling may directly modulate VTA DAergic neuronal responses via KOR vs. MOR-dependent signaling mechanisms, a previous report has demonstrated that MOR-associated VTA DAergic populations preferentially project to the BLA, while KOR-associated DAergic neurons show more specific projections to NAc targets (86). Thus, one possibility is that intra-PLC CB1 transmission may serve to shunt intra-VTA reward vs. aversion transmission through distinct efferent pathways from the VTA; a VTA → NAc, KOR aversion pathway, vs. a VTA → BLA, MOR-dependent reward. Such a model would be consistent with previous findings demonstrating that DAergic signaling within the NAc is involved importantly in mediating motivationally aversive behaviors, such as those associated with nicotine (88, 92), and findings demonstrating that DAergic transmission within the BLA is responsible for modulating opiate-related reward salience (47, 89, 93). Furthermore, these findings (78) are consistent with previous reports demonstrating that genetic deletion of the CB1 receptor renders mutant mice unresponsive to the rewarding and dependence-producing properties of opiates (65). Interestingly, systemic administration of CB1 antagonists has been reported to increase expression levels of spinal KOR substrates, and a resulting potentiation in KOR-mediated analgesic responses (94). While these studies used non-localized administration or analyses of MOR or KOR-mediated responses following systemic CB1 receptor manipulations, they further point to functionally convergent pathways mediating CB1 and opiate-receptor-specific motivational and behavioral phenomena. Importantly, the ability of cortical CB1 signaling to modulate reward processing by acting on specific opiate-receptor substrates localized to sub-cortical reward circuits, may point to another common point of convergence resulting from aberrant ECB transmission in the cortex, linking these abnormalities not only to disturbances in aversive emotional information processing, but to abnormal reward-related learning and memory, commonly seen in schizophrenia patient populations (15, 95).

## Summary and Future Directions

**Figure 3:**
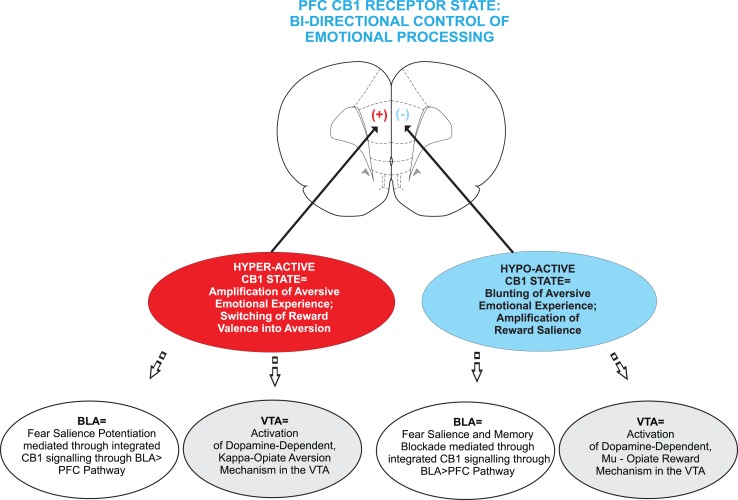
**Schematic summary of the effects of hyperactive vs. hypoactive CB1 receptor states in the mammalian PFC**. While a state of CB1 hyperactivity is linked to an amplification in emotional salience, particularly in terms of increased sensitivity to fear-related stimuli in rats and increased paranoid psychosis in human subjects. CB1 receptor overstimulation also switches normally rewarding stimuli into aversive effects, mediated through down-stream signaling of KOR receptor substrates in the VTA. In contrast, a state of CB1 hypoactivity is linked to the blunting of emotional salience, a blockade of fear-related memory formation. In contrast, CB1 receptor blockade potentiates the reward salience of opiate-related cues, via the activation of a MOR receptor substrate in the VTA.

As reviewed above, a diverse and convergent body of evidence now suggests that disturbances in ECB signaling within mesocorticolimbic circuitry may underlie sub-cortical DAergic dysregulation linked to a variety of neuropsychiatric disorders such as addiction and schizophrenia. Specifically within PFC circuits, CB1 receptor transmission appears to control bi-directional modulation of emotional salience, both in the context of aversive, negative events, and rewarding, appetitive motivational stimuli (Figure 3). The underlying mechanisms controlling these bi-directional effects of cortical ECB signaling are only now beginning to be elucidated. As the evidence presented above highlights, schizophrenia-related psychopathology likely involves core deficits in emotional processing and memory formation related to both aversive, negative stimuli and in terms of aberrant reward processing, indicated by high rates of substance abuse co-morbidities in these patient populations. Nevertheless, many critical questions remain. For example, does cortical hyperactivity of CB1 receptor substrates amplify the aversive emotional salience of normally non-salient fear stimuli via interactions with DAergic substrates in the mesocorticolimbic pathway and might these mechanisms share common interactions with opiate-receptor motivational signaling pathways in the mesolimbic circuitry?

In addition, some intriguing comparisons between the pro-psychotic, acute effects of cannabis vs. the longer term effects of cannabis has shown interesting differences in terms of their presumed effects on DAergic transmission. For example, as previously discussed, post-mortem analyses of brain tissue from human schizophrenia patients has demonstrated profound increases in CB1 receptor binding; an effect specifically evident in paranoid schizophrenia sub-groups (25, 96). In contrast, recent clinical findings have demonstrated significantly diminished DA synthesis capacity in regular marijuana users compared with non-users (97) and evidence of attenuated DA receptor expression levels in current or recently abstinent marijuana users (98, 99). However, hypo-DAergic effects of cannabis use would seem functionally contradictory to prior evidence suggesting pro-psychotic effects of cannabis exposure, effects that are generally posited to hyper-DAergic states. Furthermore, hypo-DAergic states would seem incongruent with reports demonstrating increased sensitivity to the addictive properties of opiates, particularly following chronic adolescent THC exposure (73, 75). While further studies are required to more fully address and corroborate these mechanisms, it is likely that increasing evidence will point to dysregulation in cortical ECB signaling substrates and their down-stream functional interactions with mesocorticolimbic circuits as critical underlying variables related to a variety of neuropsychiatric conditions, including schizophrenia-related psychoses, addictions, and their associated emotional processing, learning, and memory deficits.

## Conflict of Interest Statement

The authors declare that the research was conducted in the absence of any commercial or financial relationships that could be construed as a potential conflict of interest.
